# Femtosecond laser directed fabrication of optical diffusers†
†Electronic supplementary information (ESI) available: 3D view images of resulting surface topographies, and images of microstructure of ripples from rough surfaces. See DOI: 10.1039/c7ra00109f


**DOI:** 10.1039/c7ra00109f

**Published:** 2017-03-24

**Authors:** Tawfiq Alqurashi, Pavel Penchev, Ali K. Yetisen, Aydin Sabouri, Rayan M. Ameen, Stefan Dimov, Haider Butt

**Affiliations:** a Nanotechnology Laboratory, School of Engineering, University of Birmingham, Birmingham, B15 2TT, UK. Email: h.butt@bham.ac.uk; Tel: +44(0) 1214158623; b Department of Mechanical Engineering, School of Engineering, Shaqra University, Dawadmi, Saudi Arabia. Email: talqurashi@su.edu.sa; c Harvard-MIT Division of Health Sciences and Technology, Massachusetts Institute of Technology, Cambridge, Massachusetts 02139, USA; d School of Metallurgy and Materials, University of Birmingham, Birmingham, B15 2TT, UK

## Abstract

Optical diffusers are widely used in filament lamps, imaging systems, display technologies, lasers, and Light Emitting Diodes (LEDs). Here, a method for the fabrication of optical diffusers through femtosecond laser machining is demonstrated. Float glass surfaces were ablated with femtosecond laser light to form nanoscale ripples with dimensions comparable to the wavelength of visible light. These structures produce highly efficient and wide field of view diffusers. The machined patterns altered the average surface roughness, with the majority of particles in the range of a few hundred nanometers. The optical diffusion characteristic and a maximum diffusion angle of near 172° was achieved with optimum machining parameters. The transmission performance of the diffusers was measured to be ∼30% across the visible spectrum. The demonstrated technique has potential for producing low-cost large area optical devices. The process benefits from the flexibility of the laser writing method and enables the production of custom optical diffusers.

## Abstract 2

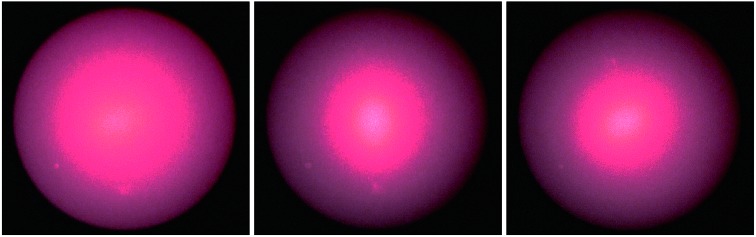
Production of optical diffusers *via* femtosecond laser based texturing of glass.

## Introduction

Optical diffusers are widely used in myriad optical devices including liquid crystal displays (LCDs), light-emitting diodes (LEDs), light sources, and lasers.^1^,^2^ Optical diffusers may consist of incorporated particles or the microstructured surface-relief patterns.^3^–^5^ The most widely used surface-relief structures include microlenses,^6^ pyramids,^7^ and textured surfaces.^8^,^9^ Applications of these types of diffusers can be found in filament lamps,^10^ LEDs^11^ and fiber optics.^12^ Fabrication of textured surfaces through femtosecond (FS) laser ablation is a rapidly growing area of interest.^13^–^15^ This fast and potentially viable method can produce micro- and nanoscale ripples on substrate surfaces to change their physical properties.^16^ Such surface texturing alters optical reflection/transmission properties.^17^

The fabrication of optical diffusers by femtosecond laser sources have not been exploited as it has challenges as compared to nanosecond pulsed lasers that exhibit excessive burrs due molten material.^18^ Direct laser processing received intensive research as result of fast growing optoelectronic market.^19^–^21^ Additionally, FS laser patterning is a deterministic process. First, it has limited thermal effect because the laser pulse ends before the electrons thermally excite any ion and also the heat diffusion outside the focal area is minimized. This leads to reduced heating caused by expansion effects and an increase in its patterning precision as compared to other laser sources.^22^,^23^

Here, we report the development of glass-based optical diffusers *via* FS laser patterning. FS laser ablation was used to produce both nano- and microstructures (∼20 μm) to increasing the roughness of the glass surfaces, which randomly diffused the transmitted light. The present method differs from other techniques used for producing diffusers, where a pulsed laser is used for patterning a photoresist layer on glass or plastic substrate.^24^ Optical scattering from the textured glasses substrate was examined by angle-resolved spectral measurements. The optical response was characterized in the visible spectrum.

## Results and discussion

### Femtosecond laser writing of glass diffusers

**Fig. 1:**
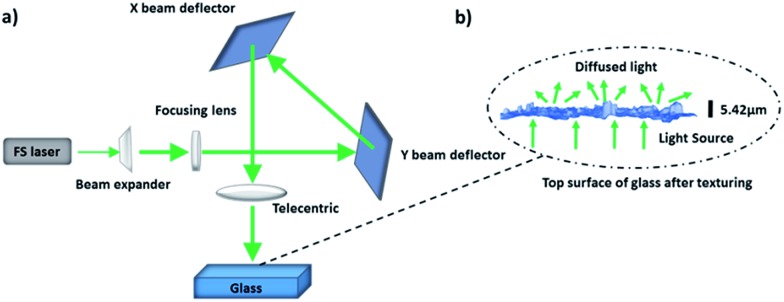
Fabrication of optical diffusers *via* FS laser patterning. (a) Schematic of the laser setup. (b) Textured surface and the resulting light diffusion.

Float glass slides were textured using an in house FS laser microprocessing platform. The surface structures on glass substrates were produced by controlling the polarization and hatch directions of the laser beam. Fig. 1a shows the schematic of the laser machining setup that includes an ultrashort ytterbium-doped fiber laser (0.8 W, 1030 nm, 310 fs, 100 kHz), a telecentric lens with a focal length of 10.0 cm and a 3D optical beam deflection system for dynamically scanning surfaces. The working envelope defined by the scan head and the focusing lens was 35 mm (*x*) × 35 mm (*y*) × 6 mm (*z*). Furthermore, the focal beam spot diameter was ∼30 μm. The glass substrate was scanned at a speed of 200 mm s^–1^ with a hatch distance of 4 μm while the pulse energy was 8 μJ. Fig. 1b shows a typical surface morphology resulting on the substrate after the FS laser processing.

**Fig. 2:**
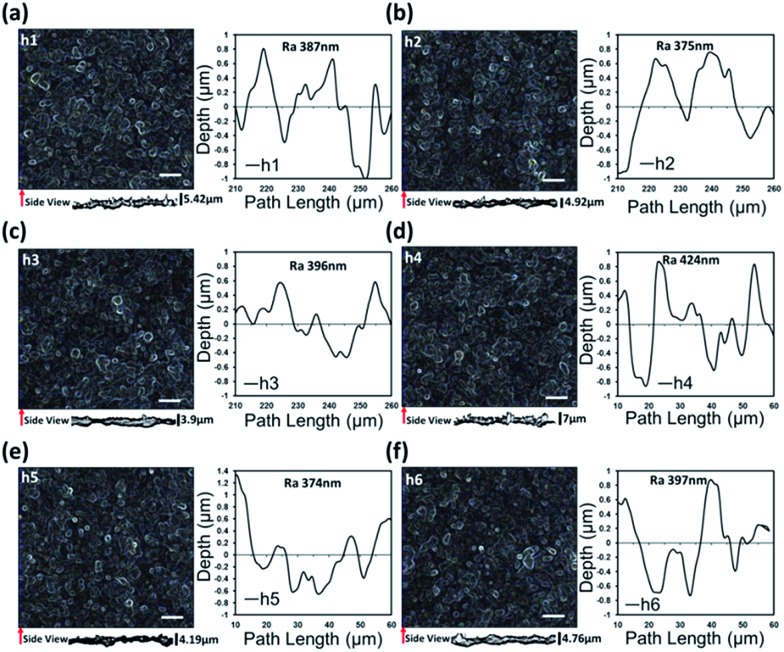
Femtosecond laser patterning of glass diffusers. Microscopic images from the top and side views of the microstructured ripples and corresponding surface roughnesses. (a–c) Samples from h1 to h3 were textured by using circular polarization with three different hatch direction, *i.e.* horizontal, vertical and at 45°, while the polarization was changed to linear for (d–f) samples from h4 to h6.

Laser parameters were varied to pattern glass substrates (Fig. 2). The surface morphology of textured glass was analyzed by an optical microscope and average roughness (*R*_a_) was measured using Alicona G5 Infinite Focus (IF) system. In Fig. 2a–c, the samples represented by h1–3 were textured using circular polarization with varying hatch directions: horizontal, vertical and at 45°, respectively. Fig. 2a–c shows the surface roughness of the fabricated diffusers along with the cross-sectional images. Side views of diffusers illustrate the difference in the surface topography produced by various laser processing parameters (hatch direction, polarization). The morphologies and arrangements of both nano- and microscale structures were dependent on the FS laser processing settings such as hatch direction.^25^,^26^ Fig. S1† shows the 3D map of the fabricated nanoripples.

Another set of samples (h4–6) were produced by changing the laser polarization to linear (Fig. 2d–f). The size and number of surface structures were reduced when the hatch direction was vertical. In general, the surface structures on all samples had non-uniform overlaps. The average surface roughness (*R*_a_) was between 375 to 424 nm (Fig. 2). The vertical hatch direction produced the lowest average roughness of 375 and 374 nm for samples h2 and h5, respectively.

**Fig. 3:**
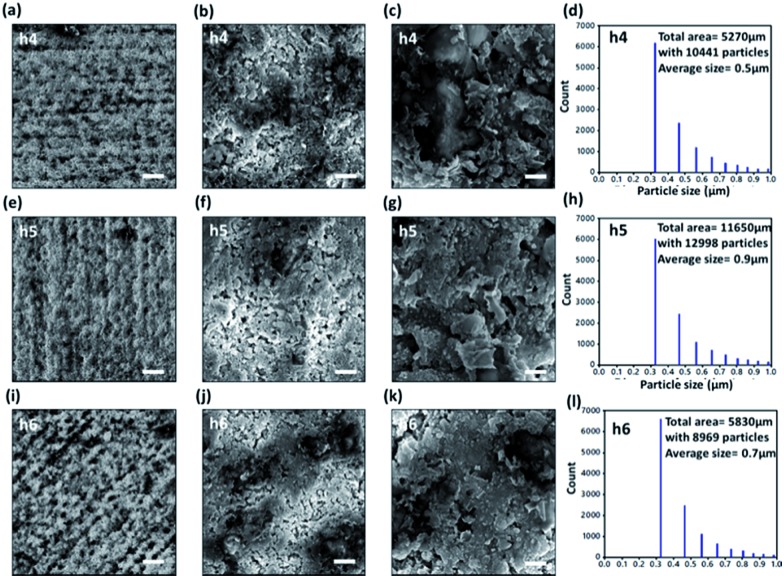
SEM images of the laser-patterned nanoripples (h4, h5 and h6). (a, e and i) 1000× magnification. Scale bar = 25 μm. (b, f and j) 20 000× magnification. Scale bar = 2.5 μm (c, g and k) 35 000 magnification. Scale bar = 1 μm. (d, h and l) Histograms showing the particle size distribution of h4, h5 and h6 samples.

The fabricated surface structures were imaged by a scanning electron microscope (SEM) (Fig. 3). Glass samples were coated with gold layer (5–10 nm). The SEM imaging was performed with a field emission gun (FEG) at 10 kV. The micro and nanoripples like structures were fabricated by the overlapping of multiple laser passes. The arrangements of structures produced were highly dependent on the laser hatch direction (Fig. 3). The high magnification images revealed that the fabrication process produced nanoscale particles on the ripples, with dimensions in the order of few hundred nanometres. These surface structures cause the scattering of light.^27^ To characterize the size of micro and nanoparticles, statistical analysis was carried out the SEM images taken from normal incidence (Fig. 3a, e and i). The grayscale SEM images were converted to binary black and white versions. Then image processing software was utilized to analyse the particles from the binary images. The particle size distribution analysis in (Fig. 3d, h and l) shows that the majority of particles have average sizes in range of 325 nm. The result matches with average roughness measurement in Fig. 2. Based on the number of particles per unit area, it can be observed that there is at least on particle per square micrometre. The highest number of particles was recorded for the h4 sample and the least for h5 sample. These particles size distribution can greatly affect the efficiency of light scattering and absorption. This gives rise to energy losses based on Rayleigh scattering and to soften light after it passing through.

### Characterization of optical diffusers

**Fig. 4:**
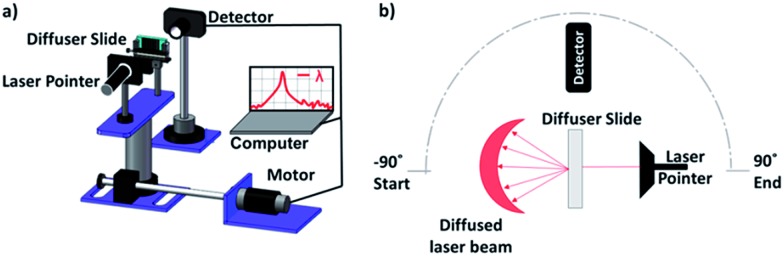
Angle-resolved spectral measurement setup for the analysis of optical diffusers. (a) Schematic of the experimental setup. (b) Top view of the experimental setup.

The samples were examined by angle-resolved optical power intensity measurements and spectroscopy analyses. The angular measurement setup consisted of a computer-controlled rotation stage, laser sources (*λ* = 633, 533, and 450 nm), and an optical powermeter (Fig. 4a). The diffuser samples were rotated with 1° increments. To reduce the stray light effect, a cylindrical tube was placed in front of the detector of the powermeter. The distances between the laser source-sample and sample-detector were 3 and 13 cm, respectively. The sample was rotated from –90° to +90° from the normal incident (Fig. 4b). The motorized stage was controlled by a software that recorded the powermeter readings at each angle simultaneously.

**Fig. 5:**
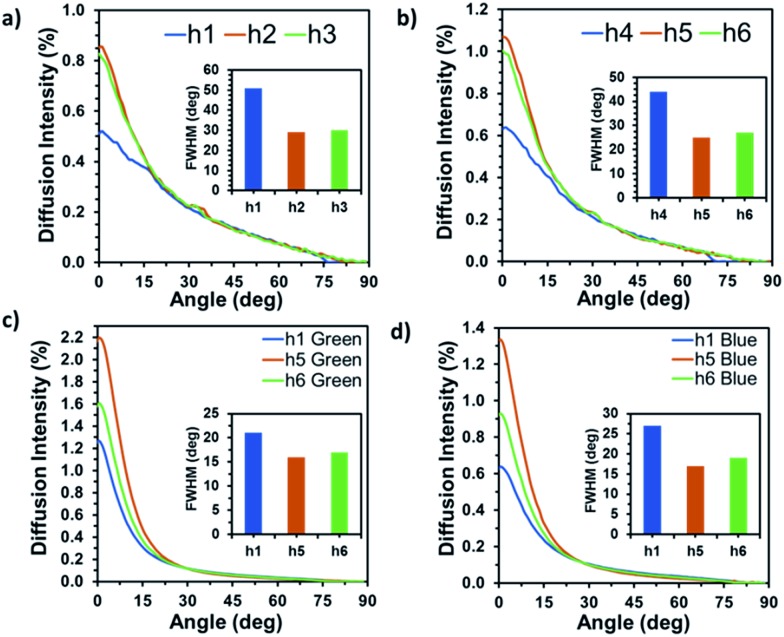
Angle-resolved measurements of the optical diffusers. (a and b) h1–6 samples using 633 nm laser beam. The insets show the full width half maxima. The diffusion in response to (c) 533 nm and (d) 450 nm laser beams.

Fig. 5a–d shows the angle-resolved measurements of transmitted diffused light. In Fig. 5a and b, measurements were performed on samples with circular and vertical polarization using a red laser beam. Fig. 5c and d shows the diffusion measurements using green and blue laser beams. The surface features produced on glass surfaces were randomly distributed causing the diffusion of light in wide angles. According to Bragg law the large diffusion angles suggest that the structures are of the same scale as the wavelength of light (few hundred nanometres). Due to the random distribution of surface structures, the nanoparticles revealed the scattering effects based on Rayleigh scattering, where the nanoparticles were smaller than the wavelength of incident light.^28^ The measured diffusion angle varied from 152° to 172°. Fig. 5a and b shows a wide diffusion angle (∼172°) with a peak transmission intensity (∼1.1%) at 0°. Fig. 5a shows that the normal transmission intensities for h1–3 were 0.5, 0.85 and 0.8% and the diffusions angles were 154°, 170° and 166°, respectively. The samples created with linear polarization (h4–6) displayed slightly higher transmission intensities (∼0.054%). Meanwhile, the diffusion angle decreased by ∼8° for samples h4 and h5. This signifies that the structures produced with linear polarization had larger lateral dimensions and lower spatial density, which led to narrower diffusion field of view and more light being focused near 0°, the diffusion properties of the samples were also analyzed using 450 and 533 nm laser beams. The samples displayed higher optical transmission for these two wavelengths (2.2% for 533 nm and 1.35% for 450 nm); however, the light scattering to higher angles was lower, 144° for 533 nm and 152° for 450 nm. Additionally, Fig. 5a–d shows the full width at half maximum (FWHM) for the diffusion plots. The highest FWHM were recorded in response to the red light; however, the intensity peak was the lowest as compared to other wavelengths. The reason being that the light was three dimensionally diffused at larger angles, hence reducing the light focused near zero degrees.

**Fig. 6:**
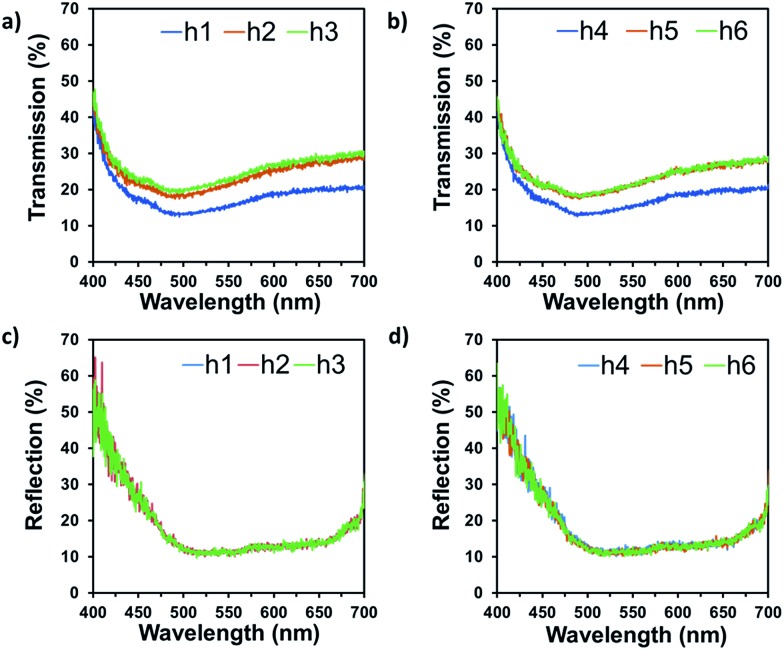
Spectroscopic measurements of the optical diffusers using broadband light, at normal incidence. (a and b) Transmission and (c and d) reflection modes.

In practical applications, diffusers are generally used for scattering white light. Hence, optical reflection and transmission measurements were also conducted using broadband light. These measurements were performed by using a spectrophotometer (2 nm resolution), which was connected to an optical microscope through an optical fiber. Fig. 6 shows the measured average reflection and transmission spectra at normal incidence. In general, the surface features reduced most of the normal light transmittance with haze boosting effects.^29^ The light is scattered at large angle and is not captured by the fibre lens placed for normal transmission measurements. The results show that ∼40% of the light was transmitted through the optical diffusers at 400 nm, and it sharply reduced to 20% at 475 nm (Fig. 6a and b). However, the transmission intensities gradually increased from 20% to 30% between 500 nm to 700 nm. The reflection measurements showed that ∼55% of the light was back-scattered from the textured surfaces at 400 nm and it reduced to 10% at 500 nm (Fig. 6c and d). For larger wavelengths, the transmission remained stable until it reached 670 nm and then increased to 27% at 700 nm. In general, the transmission and reflection measurements through the microscope setup had higher efficiency as compared to the angle-resolved measurements. This was due to the fact the microscope objectives (due to high numerical aperture) received and focussed much more light (including the diffused light) coming from the samples. To improve the efficiency, anti-reflection coating can be applied to surface to reduce reflection, as the average surface reflection is around 15% (Fig. 6c and d).

**Fig. 7:**
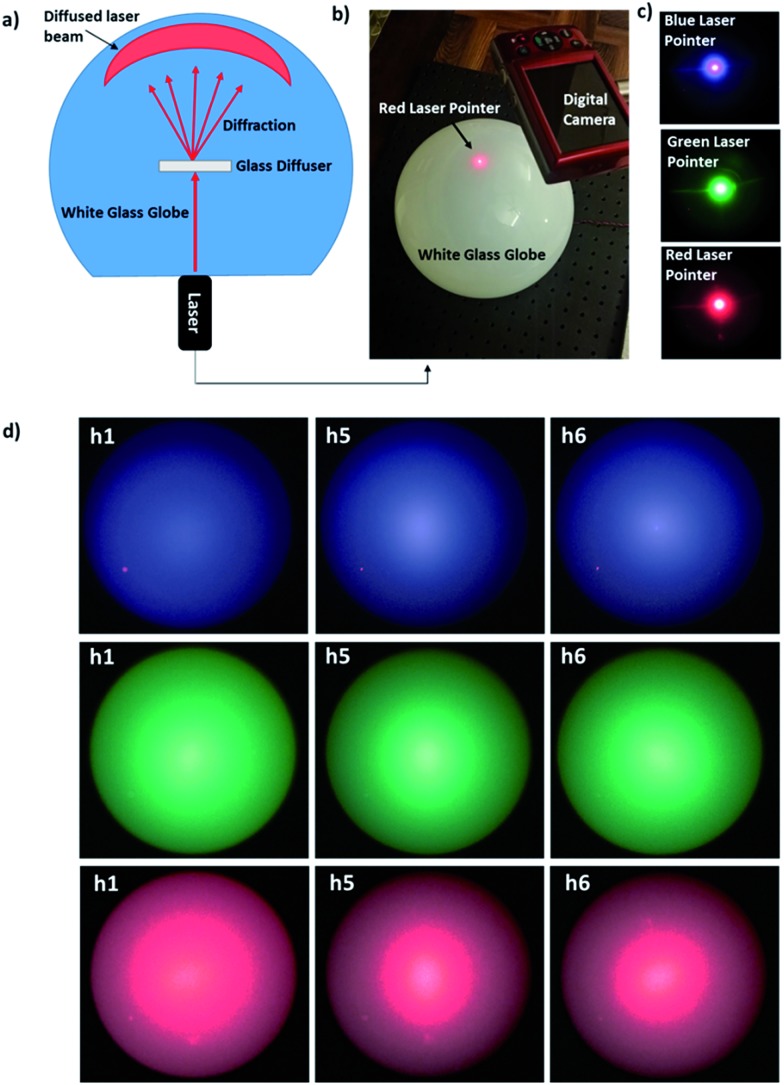
Visualization of the optical diffusion under a semi-transparent white glass globe. (a) Schematic of experimental setup used to capture the speckle patterns from the optical diffusers. (b) A photograph of experimental setup. Scale bar = 12 cm. (c) 450, 533 and 633 nm beams illuminating the glass globe without a diffuser. Scale bar = 12 cm. (d) Laser beams were passed through the diffusers producing different speckle patterns and intensities. Scale bar = 6 cm.

To visualize the three-dimensional distribution of the diffused light, the diffusers were located at the centre of a semi-transparent globe and the laser source was positioned below it (Fig. 7a–c). Fig. 7b shows photographs of the laser lights at 450, 533 and 633 nm without an optical diffuser. When the laser beams were transmitted through the optical diffusers, the light was scattered at large angles (Fig. 7d). The field view of optical diffusion increased from blue to red beams. This is in agreement with Bragg's law,^30^,^31^ which states that the diffraction angles increases for the increase in incident wavelength. Our approach showed higher diffusion angles as compared to the commercial polished and sand-blasted diffusers, where glass diffuser achieves light scattering up to 40°. In addition our fabrication is very economical for scale up manufacturing, due to fast laser writing speeds, flexible and tailored pattering of glass substrates.^2^,^4^,^32^

## Conclusions

We have developed a cost effective method for fabricating optical diffusers by using femtosecond laser ablation. The laser machining nano and microscale surface structures on the float glass increased the diffusion angle up to ∼172° and reduced light normal transmittance. The effects of hatch direction and laser polarization on transmission characteristics of the diffusers were also investigated. The maximum and minimum transmission intensities were at 400 and 475 nm, respectively. The fabrication method developed in this work has fast processing time and flexibility of patterning various geometries. Optical diffusers produce by this method may have applications in photonic communication systems, displays, and LEDs.

## Conflict of interest

The authors declare no competing financial interests.

## Supplementary Material

Supplementary information
