# Supplementary material for: Femtosecond laser directed fabrication of optical diffusers
Source: RSC Adv. 2017 Mar 24;7(29):18019–23. doi: 10.1039/c7ra00109f (PMC6112377; doi:10.1039/c7ra00109f)
Supplement: Supplementary file 1 [file RA-007-C7RA00109F-s001.pdf]

Electronic Supplementary Material (ESI) for RSC Advances.
This journal is © The Royal Society of Chemistry 2017

SUPPORTING INFORMATION

Femtosecond Laser Directed Fabrication of Optical Diffusers

Tawfiq Alqurashi[1,2,ƚ], Pavel Penchev [1], Ali K. Yetisen[3], Aydin Sabouri, [1]Rayan M. Ameen [4], Stefan Dimov[1], and Haider Butt[1,]*

[1]School of Engineering, University of Birmingham, Birmingham, B15 2TT, UK

[2]Department of Mechanical Engineering, School of Engineering, Shaqra University, Dawadmi, Saudi Arabia

[3]Harvard-MIT Division of Health Sciences and Technology, Massachusetts Institute of Technology, Cambridge, Massachusetts 02139, USA

[4]School of Metallurgy and Materials, University of Birmingham, Birmingham, B15 2TT, UK

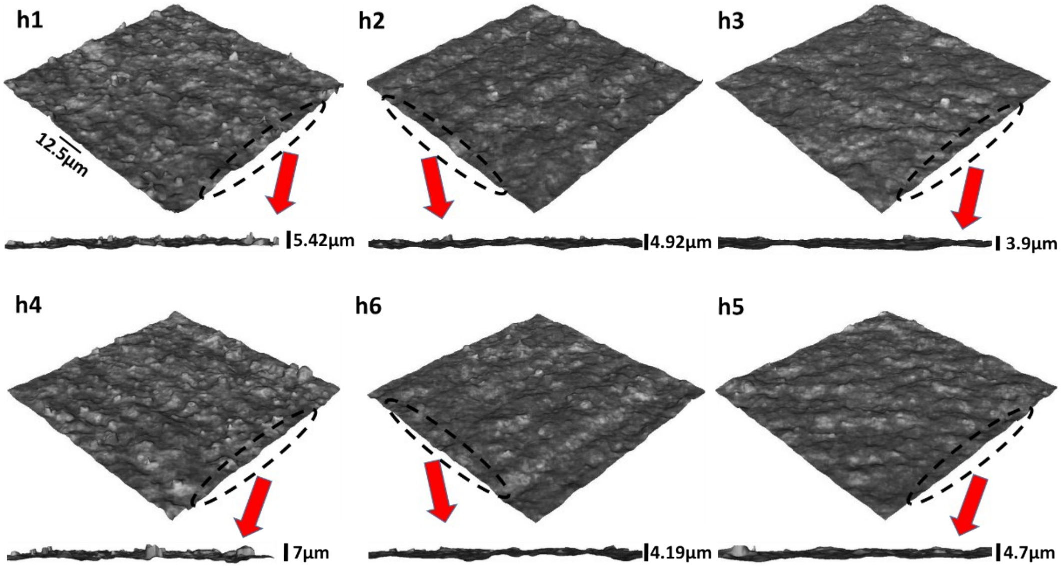

Fig. S1. 3D view images of resulting surface topographies were captured by Alicona system.

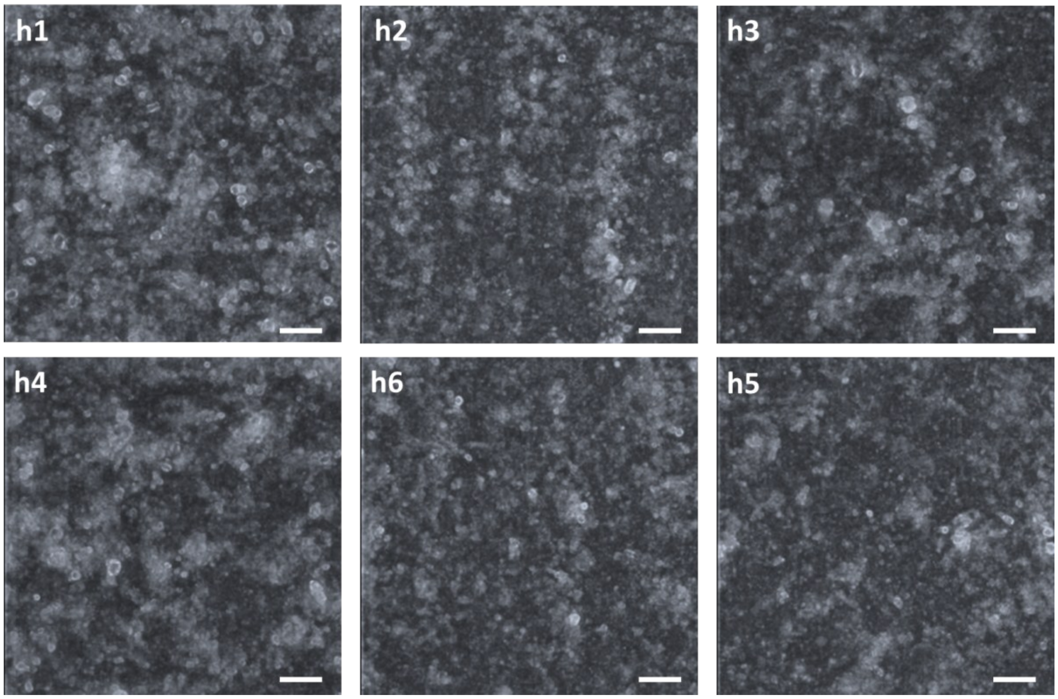

Fig. S2. Normal view images of microstructure of ripples from rough surfaces were captured by Alicona system. Scale bar= 10 µm.
